# RECQL5 and BLM exhibit divergent functions in cells defective for the Fanconi anemia pathway

**DOI:** 10.1093/nar/gku1334

**Published:** 2014-12-17

**Authors:** Tae Moon Kim, Mi Young Son, Sherry Dodds, Lingchuan Hu, Guangbin Luo, Paul Hasty

**Affiliations:** 1 Department of Molecular Medicine and Institute of Biotechnology, The Barshop Center of Aging, University of Texas Health Science Center, San Antonio, TX 78245, USA; 2 Department of Genetics, Case Western Reserve University, BRB-720, 10900 Euclid Avenue, Cleveland, OH 44106, USA

## Abstract

Fanconi anemia (FA) patients exhibit bone marrow failure, developmental defects and cancer. The FA pathway maintains chromosomal stability in concert with replication fork maintenance and DNA double strand break (DSB) repair pathways including RAD51-mediated homologous recombination (HR). RAD51 is a recombinase that maintains replication forks and repairs DSBs, but also rearranges chromosomes. Two RecQ helicases, RECQL5 and Bloom syndrome mutated (BLM) suppress HR through nonredundant mechanisms. Here we test the impact deletion of RECQL5 and BLM has on mouse embryonic stem (ES) cells deleted for FANCB, a member of the FA core complex. We show that RECQL5, but not BLM, conferred resistance to mitomycin C (MMC, an interstrand crosslinker) and camptothecin (CPT, a type 1 topoisomerase inhibitor) in FANCB-defective cells. RECQL5 suppressed, while BLM caused, breaks and radials in FANCB-deleted cells exposed to CPT or MMC, respectively. RECQL5 protected the nascent replication strand from MRE11-mediated degradation and restarted stressed replication forks in a manner additive to FANCB. By contrast BLM restarted, but did not protect, replication forks in a manner epistatic to FANCB. RECQL5 also lowered RAD51 levels in FANCB-deleted cells at stressed replication sites implicating a rearrangement avoidance mechanism. Thus, RECQL5 and BLM impact FANCB-defective cells differently in response to replication stress with relevance to chemotherapeutic regimes.

## INTRODUCTION

Genetic mutations in the Fanconi anemia (FA) pathway cause bone marrow failure, developmental defects, cancer, hypersensitivity to DNA interstrand crosslinks and chromosomal instability (1). Even though FA is rare, loss of FA function strongly correlates with metastasis and poor prognosis in sporadic breast cancer (2). Many proteins constitute the FA pathway and are categorized into three groups (1,3). Group 1 proteins form a core complex that identifies DNA damage. The FA core complex monoubiquitinates FANCD2 (4) to enable activation of the group 2 proteins: FANCD2 and FANCI (5). Group 3 proteins are not required for FANCD2 monoubiquitination but instead orchestrate other pathways required for efficient double strand break (DSB) repair.

Homologous recombination (HR) and nonhomologous end joining (NHEJ) are nonredundant pathways important for DSB repair (6). NHEJ repairs DSBs in both G_1_ and S/G_2_ by simply joining free ends. A key component includes the KU heterodimer composed of KU70 and KU80 that binds DNA ends (7). In FA-defective cells, KU70-deletion improved resistance and reduced chromosomal alterations after exposure to crosslinking agents suggesting that the FA pathway diverts DSB repair from NHEJ to HR (8). HR maintains chromosomal integrity through DSB repair and replication forks maintenance. For DSB repair, the RAD51 recombinase nucleates onto 3′ single DNA strand ends to initiate invasion to a homologous template, usually provided by the complementary sister chromatid during replication (9). RAD51 also protects the nascent DNA strand to enhance continuous replication and reduce the number and size of single strand gaps (10) and stabilizes replication forks and enables replication fork restart (11–17). RAD51 is linked to FA since it associates with the FA proteins BRCA2 (18,19), FANCD2 (20) and RAD51C (21). Furthermore in FA-defective cells, BRCA2 stabilization of the RAD51 filament protected replication forks from MRE11 exonuclease activity that is required to initiate HR (11,12). BRCA2 is an FA group 3 protein (a.k.a. FANCD1) and functionally interacts with FANCD2 (22). Thus, the FA pathway is genetically integrated with NHEJ and functionally integrated with HR.

The RecQ helicases, RECQL5 and Bloom syndrome mutated (BLM) regulate HR to suppress rogue recombination (23) through nonredundant mechanisms (24). RECQL5 shunts the repair of DSBs to synthesis-dependent strand annealing (SDSA) by disrupting RAD51 nucleoprotein filaments (25,26) while BLM inhibits crossing over through Holliday junction dissolution (27). *Recql5* and *Blm* were mutated in mouse embryonic stem (ES) cells (24). Reduction of either protein increased levels of sister chromatid exchanges (SCEs) and increased gene targeting (24,28) and their combined reduction further elevated SCEs demonstrating these proteins are not redundant or epistatic (24). In addition, the FA core complex associates with a BLM supercomplex called BRAFT (1). BLM colocalizes with FANCD2 and the FA core complex is required for BLM phosphorylation and nuclear foci formation in response to interstrand crosslinks (ICLs) (29). Just how RECQL5 and BLM influence the FA phenotype is not known at a biological level.

FA and HR are integrated in response to various replication fork-blocking agents in a manner that is not fully understood. Some agents physically interfere with separating DNA strands to block replication fork progression like mitomycin C (MMC) and camptothecin (CPT). MMC is a bifunctional alkylating agent that forms monoadducts, intra- and interstrand crosslinks (30). Interstrand crosslinks are the most deleterious since they tether complimentary strands and cause DSBs after collision with a replication fork (31). CPT is a type 1 topoisomerase (topo 1) inhibitor that stabilizes a ternary complex between topo 1 and double-stranded DNA resulting in single strand breaks that become DSBs at replication forks (32). In addition, topo 1 depletion increases positive supercoils ahead of the replication fork to induce fork regression (a chicken foot) (33–35). Thus, MMC and CPT cause a diversity of challenges to replication fork progression.

To investigate the genetic and functional integration between HR regulators and the FA pathway, we mutated *FancB* in *Recql5*- and *Blm*-mutant mouse ES cells (24,28). FANCB is an essential member of the FA core complex (36) that is capable of monoubiquitinating FANCD2 in a minimal subcomplex with two other FA core complex proteins, FAAP100 and FANCL (4). Disruption of this catalytic module completely destroys core complex function (37). Previously we reported that cells deleted for *FancB* exon 2 (*fancb^Δex2^*) exhibited a typical FA phenotype that included reduced cellular proliferation, increased MMC sensitivity, increased spontaneous and MMC-induced chromosomal abnormalities, reduced MMC-induced RAD51 foci and absent MMC-induced FANCD2 foci (38). Here we show that deletion of RECQL5, but not BLM, enhanced toxicity to MMC and CPT in *fancb^Δex2^* cells. RECQL5-deletion enhanced CPT-induced chromosomal instability while BLM-deletion suppressed MMC-induced chromosomal defects. Furthermore, in *fancb^Δex2^* cells, deletion of RECQL5, but not BLM, exacerbated a defect in replication fork protection/restart. RECQL5 also suppressed RAD51 levels at stressed replication forks. These studies suggest that RECQL5 deletion would enhance sensitivity, while BLM deficiency would enhance resistance, to chemotherapeutics for FA-deficient cancers.

## MATERIALS AND METHODS

### Tissue culture conditions and cells

ES cells were maintained on 0.1% gelatin-coated plastic plates in a 37°C incubator at atmospheric O_2_ in Minimal Essential Media-α (Invitrogen/Gibco, Carlsbad, CA, USA) with 15% fetal bovine serum (Invitrogen/Gibco, Carlsbad, CA, USA), 2-mM glutamine, 30-μg penicillin/ml, 50-μg streptomycin/ml, 10^−4^-M β-mercaptoethanol and 1 X leukocyte inhibiting factor (Gemini Bio products).

We observed mouse ES cells mutated for *FancB, Blm* and *Recql5*. All these cells are in an AB2.2 background. *Blm* deletion is lethal so we use cells with a complicated mutation that ultimately reduces protein levels by ∼88% (28,39). These mutant cells are called *blm^tm3Brd/tm4Brd^* but, in this proposal, are simply called *blm^−/−^*. We used *Recql5*-mutant cells deleted for exon four, called *recql5^−/−^* (24). *FancB* exon 2 was deleted in *blm^−/−^* and *recql5^−/−^* cells as described (38).

### Cell cycle analysis

FACScalibur and LSRII were used to perform a cell cycle analysis as described (40).

### Survival fraction

Survival fraction was performed as described (41).

### Two-color fluorescence *in situ* hybridization

Cells were seeded onto gelatin-treated (0.1%, 1 h) plastic tissue culture plates. The following day cells were exposed to MMC (30 nM, 16 h) or CPT (100 nM, 16 h), then colcemid (540 nM, 4 h) and then trypsinized to isolate cells. The rest of the procedure was performed as described (42).

### Fiber analysis

This procedure has been previously described (40). Fiber analysis with mirin treatment has been previously described (11).

### iPOND

This procedure has been previously described (40). For quantification, enhanced chemiluminescence film scans were quantified using Licor's Image Studio Lite software. IRDye fluorescent blots, Histone H3 and γH2AX only, were scanned and quantified using the Odyssey Infrared Imaging System, Application Version 3.0 software. For both software programs, a local background subtraction method was used to subtract independent background values for each sample. The background was defined as ‘median’ background with a 3 pixel width border above and below each box. Normalized ratios for each antibody were calculated against Histone H3 loading control. These ratios were then used to calculate fold change by dividing each treatment ratio against the AB2.2 control ratio.

## RESULTS

### Deletion of RECQL5 and BLM caused a different biological outcome for FANCB-deleted cells in response to replication-associated DSBs

Our goal is to evaluate the possibility that either RECQL5 or BLM impacts the FA pathway since both helicases regulate HR in a nonredundant manner and since the FA pathway is genetically and functionally integrated with HR. To achieve this goal *FancB* exon 2 was deleted in mouse ES cells (*fancb^Δex2^*) (38) previously mutated for either *Recql5* (exon 4 deletion, *recql5^−/−^*) (24) or *Blm* (*blm^tm3Brd/tm4Brd^*, simply called *blm^−/−^* for this paper) (28). The *Fancb* and the *Recql5* mutations are likely null while the *Blm-*mutation reduces protein expression by ∼88% (null is cell lethal) (28,39). All cells are in the AB2.2 genetic background.

**Figure 1.**
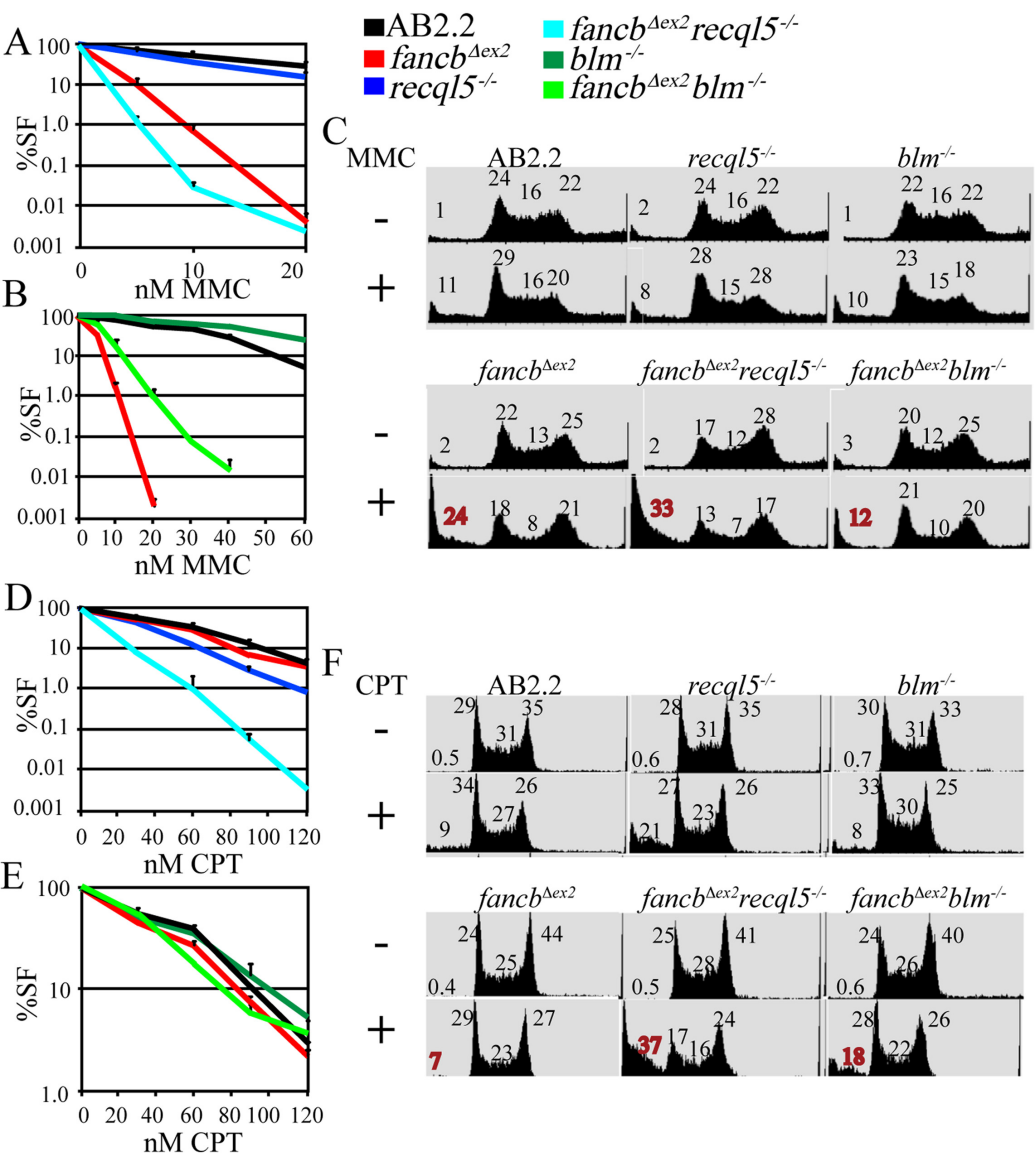
The survival fraction (SF) of *fancb^Δex2^* cells exposed to (**A–C**) MMC or (**D–F**) CPT deficient for either (A, C, D, F) RECQL5 or (B, C, E, F) BLM. The survival fractions are the average of three experiments. Flow cytometry before and 36 h after exposure to (C) MMC (10 nM 16 h) or (F) CPT (100 nM 16 h). The percentage of cells in different stages of the cell cycle is shown: the sub-G_1_ population is to the far left followed by G_1_, S and G_2_ phases.

Sensitivity to the replication stressors MMC (Figure 1A–C) and CPT (Figure 1D–F) was measured in *fancb^Δex2^* cells deleted for either RECQL5 (Figure 1A, C, D and F) or BLM (Figure 1B, C, E and F) using either a cell proliferation assay to measure survival (Figure 1A, B, D and E) (41) or a flow cytometry assay to measure cell cycle distribution and cell death (sub-G_1_ population) (Figure 1C and F). In AB2.2 control cells, FANCB-deletion increased sensitivity to MMC but not CPT, RECQL5-deletion mildly increased sensitivity to MMC and CPT (43) and BLM-deficiency did not impact sensitivity to MMC or CPT. In *fancb^Δex2^* cells, RECQL5-deletion exacerbated sensitivity to MMC and CPT while BLM-deficiency enhanced resistance to MMC and had little impact on resistance to CPT. Thus, deleting either RECQL5 or BLM had a divergent impact on *fancb^Δex2^* cells exposed to replication stressors showing a unique genetic interdependence with the FA core complex.

**Figure 2.**
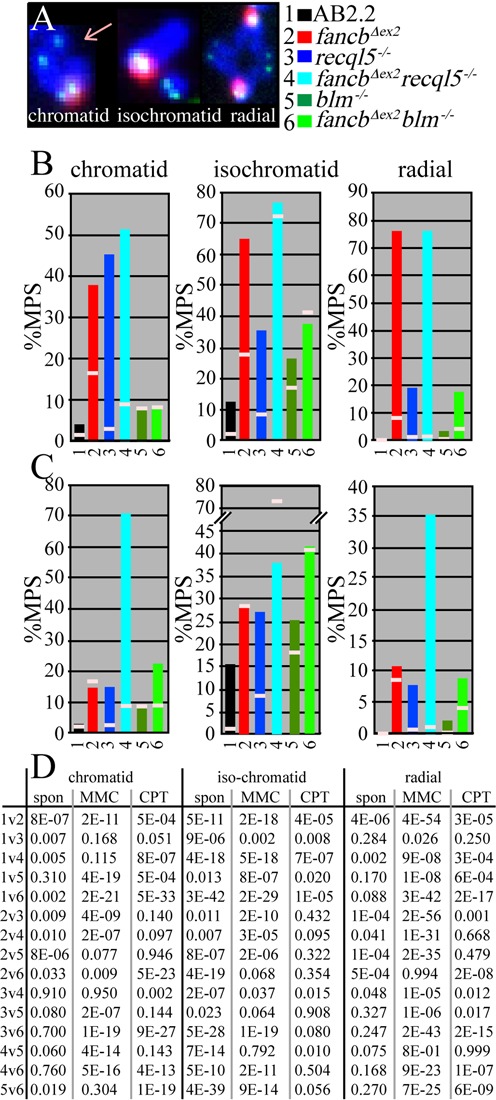
Chromosomal defects in *fancb^Δex2^* cells deleted for either *Recql5* or *Blm*. (**A**) Chromosomal aberrations. (**B**) MMC-induced chromosomal abnormalities before (rose lines) and 16 h after (colored lines in legend) exposure to 30-nM MMC. Note *Recql5*-deletion has no effect or exacerbates all MMC-induced aberrations while *Blm*-deletion ameliorates all MMC-induced aberrations for *fancb^Δex2^* cells. (**C**) CPT-induced chromosomal abnormalities before (rose lines) and 16 h after (colored lines in legend) exposure to 100-nM CPT. Note that FA deletion only increases chromatid breaks and radials in the absence of RECQL5. (**D**) Statistics for spontaneous (spon), MMC-induced and CPT-induced chromosomal abnormalities. Yates-Corrected Chi Square. Numbers same as key at top. Number of MPS observed for no exposure: AB2.2 (168), *fancb^Δex2^* (212), *blm^−/−^* (177), *fancb^Δex2^ blm^−/−^* (200), *recql5^−/−^* (210), *fancb^Δex2^ recql5^−/−^* (202). Number of MPS observed for MMC exposure: AB2.2 (166), *fancb^Δex2^* (100), *blm^−/−^* (199), *fancb^Δex2^ blm^−/−^* (151), *recql5^−/−^* (202), *fancb^Δex2^ recql5^−/−^* (141). Number of MPS observed for CPT exposure: AB2.2 (170), *fancb^Δex2^* (201), *blm^−/−^* (162), *fancb^Δex2^ blm^−/−^* (131), *recql5^−/−^* (133), *fancb^Δex2^ recql5^−/−^* (168).

We next observed chromosomal abnormalities using two-color fluorescence *in situ* hybridization. This assay uses a probe to detect telomeres (green) and another probe to detect major satellite repeats in pericentromeres (red). The chromosome arms are counterstained with 4',6-diamino-2-phenylindole (DAPI) (blue). Previously we reported that *fancb^Δex2^* cells exhibited increased levels of spontaneous and MMC-induced chromatid breaks, isochromatid breaks and radials (Figure 2A) (38). A chromatid break is a single broken chromatid that is consistent with a broken replication fork. An isochromatid break is a break in two complementary sister chromatids at the same location and is consistent with a failed SCE intermediate. A radial is the product of multiple chromosome attachments and is consistent with the fusion of broken chromatids.

Spontaneous chromosomal abnormalities were observed. The *fancb^Δex2^, blm^−/−^* and *recql5^−/−^* cells exhibited elevated levels of spontaneous chromatid breaks, isochromatid breaks and radials as compared to AB2.2 control cells (Figure 2B and C: the small pink horizontal lines that are superimposed on the vertical colored bars represent spontaneous defects; statistics shown in Figure 2D). The *fancb^Δex2^* cells exhibited more of these abnormalities than the *recql5^−/−^* and *blm^−/−^* cells. In *fancb^Δex2^* cells, mutation of either helicase suppressed chromatid breaks and radials suggesting that RECQL5 and BLM enable these defects and that chromatid breaks (diagnostic of one-ended breaks at collapsed forks) are precursors for radials. By contrast, deletion of either helicase enhanced isochromatid breaks for *fancb^Δex2^* cells, in particular RECQL5, suggesting that they ameliorate this defect and that their deficiency leaves unresolved HR-intermediates in keeping with their regulatory role.

MMC-induced chromosomal abnormalities were observed (Figure 2B; statistics shown in D). After MMC-exposure (30 nM, 16 h) in AB2.2 control cells, deletion of FANCB or RECQL5 increased the level of chromatid breaks, isochromatid breaks and radials, but BLM-deficiency caused little change. In *fancb^Δex2^* cells, RECQL5-deletion mildly increased chromatid and isochromatid breaks but did not change radials while BLM-deficiency decreased all chromosomal abnormalities. Thus, a clear distinction is revealed between RECQL5 and BLM in their relationship to the FA pathway's response to MMC-induced damage. These observations suggest BLM enables chromosomal defects in FA-defective cells exposed to MMC, similar to KU70 (8). It is possible that with MMC-induced damage, BLM-mediated Holliday junction dissolution shifts repair from HR to a more mutagenic pathway (possibly KU70-mediated NHEJ), but RECQL5-mediated RAD51 synaptic filament disassembly is irrelevant.

CPT-induced chromosomal abnormalities were observed (Figure 2C; statistics shown in D). After CPT-exposure (100 nM, 16 h) in AB2.2 control cells, RECQL5-deletion increased the levels of all abnormalities, but deficiency of FANCB or BLM had little impact. Thus, the FA pathway responds differently to lesions induced by CPT as compared to MMC even though both cause DSBs at replication forks. Since CPT inhibits type 1 topoisomerases, then supercoiling could protect against these abnormalities in FA-defective cells. Interestingly in *fancb^Δex2^* cells, deletion of RECQL5, but not BLM, increased chromatid breaks and radials; thus, only RECQL5 suppresses these biological outcomes. Similar to the spontaneous data, chromatid, but not isochromatid, breaks track with radials suggesting the former are substrates for the latter. Furthermore, these observations are consistent with the possibility that in *fancb^Δex2^* cells, RECQL5-mediated RAD51 filament disassembly shunts repair to a less mutagenic pathway (possibly SDSA). It is also possible that supercoiling suppresses unbridled HR. In support, as compared to spontaneous, CPT reduced isochromatid breaks in *fancb^Δex2^ recql5^−/−^* cells suggesting that supercoiling suppresses their causal factors; likely intractable HR intermediates. CPT might also have less impact in *blm^−/−^* cells since BLM associates with topo IIIα to dissolve Holliday junctions; thus, BLM-deletion would blunt CPT's full impact by rendering topo IIIα inactive (44).

### In *fancb^Δex2^* cells, deletion of RECQL5, but not BLM, caused an additive defect in nascent strand protection and replication fork restart

**Figure 3.**
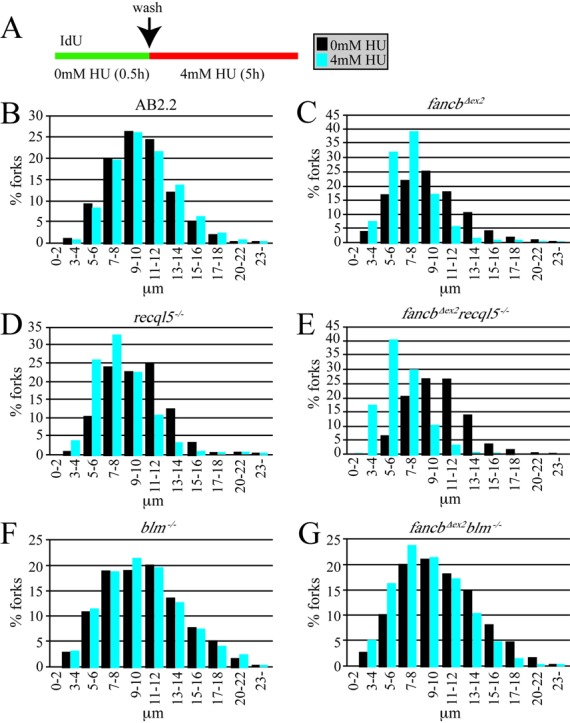
FANCB and RECQL5, but not BLM, protect the nascent strand after HU exposure in an additive manner. (**A**) Experimental conditions. (**B**) Control AB2.2 cells. (**C**) *fancb^Δex2^* cells. (**D**) *recql5^−/−^* cells (**E**) *fancb^Δex2^ recql5^−/−^* cells. (**F**) *blm5^−/−^* cells (**G**) *fancb^Δex2^ blm^−/−^* cells. Refer to Supplementary Table S1 for statistics. These data are from a single experiment. The number of fibers counted and mean fiber length for no treatment: control (1566, 9.627 μm), *fancb^Δex2^* (1206, 8.892 μm), *recql5^−/−^* (1149, 9.375 μm), *fancb^Δex2^ recql5^−/−^* (1183, 9.792 μm), *blm^−/−^* (1447, 10.915 μm), *fancb^Δex2^ blm^−/−^* (1283, 9.937 μm). The number of fibers counted and mean fiber length for HU exposed: control (1701, 9.871 μm: 0% decrease), *fancb^Δex2^* (1467, 6.711 μm: 25% decrease), *recql5^−/−^* (2336, 7.521 μm: 20% decrease), *fancb^Δex2^ recql5^−/−^* (2223, 5.886 μm: 40% decrease), *blm^−/−^* (1731, 9.930 μm: 0.85% decrease), *fancb^Δex2^ blm^−/−^* (1430, 8.700 μm: 12.4% decrease).

We investigated the impact RECQL5 and BLM have on replication fork maintenance in *fancb^Δex2^* cells after exposure to replication fork blocking agents. Cells were exposed to IdU for 30 min, then a severe hydroxyurea (HU) dose (4 mM, 5 h) that causes breaks (40) and the length of the IdU-labeled strand was measured (Figure 3A). After HU exposure, AB2.2 control cells did not exhibit nascent strand degradation (Figure 3B; refer to Supplementary Table S1, Supplementary material, for statistics). Cells deleted for FANCB (Figure 3C and Supplementary Table S1) or RECQL5 (Figure 3D and Supplementary Table S1) exhibited nascent strand degradation in an additive manner (Figure 3E and Supplementary Table S1). Consistent with previous results (12), BLM-deficient cells did not show HU-induced nascent strand degradation (Figure 3F and Supplementary Table S1). In addition, BLM-deficiency ameliorated nascent strand degradation in *fancb^Δex2^* cells (Figure 3G and Supplementary Table S1), suggesting that BLM actually enhances degradation in FA-defective cells. It is possible this activity contributes to the BLM-induced sensitivity to MMC in *fancb^Δex2^* cells shown in Figures 1B and C and 2B. Thus, FANCB and RECQL5, but not BLM, protect the nascent strand from degradation in a nonepistatic relationship.

**Figure 4.**
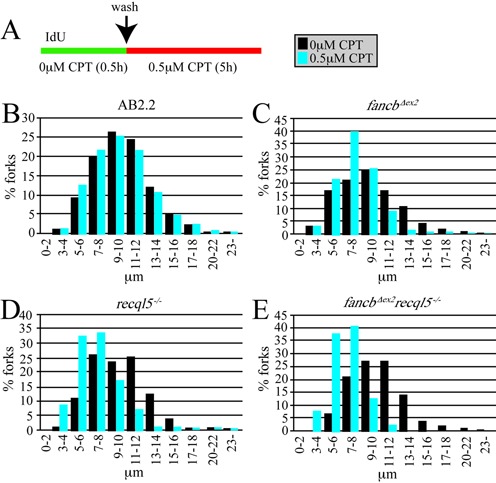
FANCB and RECQL5 protect the nascent strand after CPT exposure in an additive manner. (**A**) Experimental conditions. (**B**) Control AB2.2 cells. (**C**) *fancb^Δex2^* cells. (**D**) *recql5^−/−^* cells (**E**) *fancb^Δex2^ recql5^−/−^* cells. Refer to Supplementary Table S1 for statistics. These data are from a single experiment. The number of fibers counted and mean fiber length for no treatment is shown in the legend for Figure 3. The number of fibers counted and mean fiber length for CPT exposed: control (3096, 9.374 μm), *fancb^Δex2^* (2571, 7.466 μm: 16% decrease), *recql5^−/−^* (2264, 6.778 μm: 27.7% decrease), *fancb^Δex2^ recql5^−/−^* (2476, 6.285 μm: 35.8% decrease).

Nascent strand protection was also tested in cells deleted for FANCB and RECQL5 after exposure to CPT. Cells were exposed to IdU for 30 min, then CPT (0.5 μM, 5 h) and the length of the IdU-labeled strand was measured (Figure 4A). After CPT exposure, AB2.2 control cells did not exhibit nascent strand degradation (Figure 4B; refer to Supplementary Table S1 for statistics). Cells deleted for FANCB (Figure 4C and Supplementary Table S1) or RECQL5 (Figure 4D and Supplementary Table S1) exhibited nascent strand degradation in an additive manner (Figure 4E and Supplementary Table S1). Thus similar to HU, FANCB and RECQL5 protect the nascent replication strand from degradation after exposure to CPT in a nonepistatic relationship.

**Figure 5.**
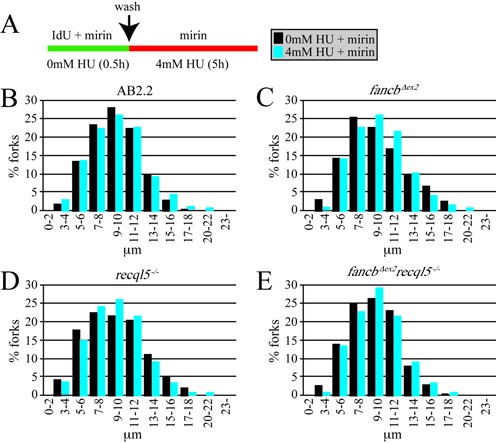
FANCB and RECQL5 protect the nascent strand from MRE11-medated degradation after HU exposure. (**A**) Experimental conditions. (**B**) Control AB2.2 cells. (**C**) *fancb^Δex2^* cells. (**D**) *recql5^−/−^* cells (**E**) *fancb^Δex2^ recql5^−/−^* cells. Refer to Supplementary Table S1 for statistics. These data are from a single experiment. The number of fibers counted and mean fiber length for no HU + mirin exposed: control (1416, 8.828 μm), *fancb^Δex2^* (1301, 9.137 μm), *recql5^−/−^* (1491, 8.817 μm), *fancb^Δex2^ recql5^−/−^* (1384, 8.727 μm). The number of fibers counted and mean fiber length for HU + mirin exposed: control (1430, 8.955 μm: 0% decrease), *fancb^Δex2^* (1256, 9.184 μm: 0% decrease), *recql5^−/−^* (1487, 8.815 μm: ∼0% decrease), *fancb^Δex2^ recql5^−/−^* (1363, 8.944 μm: 0% decrease).

BRCA2 was shown to inhibit MRE11-mediated degradation of the nascent strand at collapsed replication forks (11). Therefore, HU-induced nascent strand degradation was measured in the presence of an MRE11 inhibitor, mirin (Figure 5A). Cells were exposed to IdU + mirin for 30 min, then IdU was removed but mirin was maintained and a severe HU dose (4 mM, 5 h) that causes breaks was added (40) and the length of the IdU-labeled strand was measured (Figure 5A). Mirin negated nascent strand degradation for all genotypes (Figure 5B–E and Supplementary Table S1). Thus like BRCA2, FANCB and RECQL5 protect the nascent replication strand from MRE11-mediated degradation. A comparison between BRCA2 and RECQL5 is intriguing since the former stabilizes while the latter dissolves RAD51 filaments. This comparison suggests these two RAD51 filaments are antithetical since the BRCA2-stabilized filament protects while the RECQL5-dissolved filament assaults nascent strand length.

**Figure 6.**
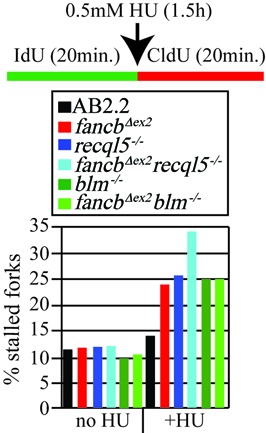
Replication fork restart for *fancb^Δex2^* cells mutated for *Recql5* or *Blm*. Refer to Supplementary Table S2 for statistics. These data are the average of a single experiment. For no treatment, the number of total fibers counted [restart, stalled and new origin]: AB2.2 control: 926 total [809 (87.37%), 100 (10.8%), 17 (1.84%)], *fancb^Δex2^*: 1497 [1304 (87.11%), 162 (10.82%), 31 (2.07%)], *recql5^−/−^*: 2137 total [1862 (87.13%), 234 (10.95%), 41 (1.92%)], *fancb^Δex2^ recql5^−/−^*:1752 [1498 (85.5%), 214 (12.21%), 46 (2.2%). *blm^−/−^*: 2135 total [1878 (87.7%), 216 (10.1%), xx (xx%)], *fancb^Δex2^ blm^−/−^*:1843 [1615 (88.6%), 194 (10.5%), 34 (1.8%)]. For HU treatment the number of fibers counted [restart, stalled and new origin]: AB2.2 control: 1809 [1516 (83.80%), 256 (13.93%), 41 (2.27%)], *fancb^Δex2^*: 2031 [1460 (71.89%), 516 (25.41%), 55 (2.71%)], *recql5^−/−^*: 2670 [1976 (74.04%), 638 (23.9%), 56 (2.1%)], *fancb^Δex2^ recql5^−/−^*: 2363 [1491 (63.1%), 798 (33.77%), 74 (3.13%)]. *blm^−/−^*: 1554 total [1136 (78.1%), 388 (25%), 30 (1.9%)], *fancb^Δex2^ blm^−/−^*:1880 [1374 (73.1%), 467 (24.8%), 39 (2.1%)].

We next observed the impact RECQL5 and BLM have on replication fork restart in *fancb^Δex2^* cells. Cells were exposed to IdU for 20 min, then a mild HU dose (0.5 mM, 1.5 h) that does not cause breaks (40) and then CldU for 20 min. AB2.2 control cells exhibited a marginal decrease in replication fork restart under these conditions (Figure 6; refer to Supplementary Table S2, Supplementary material, for statistics). Similar to nascent strand protection, deletion of RECQL5 and FANCB decreased replication fork restart in an additive manner (Figure 6 and Supplementary Table S2). Consistent with previous results (45), BLM-depleted cells were also defective in replication fork restart, yet this activity was epistatic to FANCB (Figure 6 and Supplementary Table S2), as would be predicted from its association with FANCD2 (46). Thus, RECQL5 was independent, while BLM was epistatic, to FANCB-mediated replication fork restart.

### Deletion of FANCB and RECQL5 influenced protein levels at stalled and collapsed replication forks

**Figure 7.**
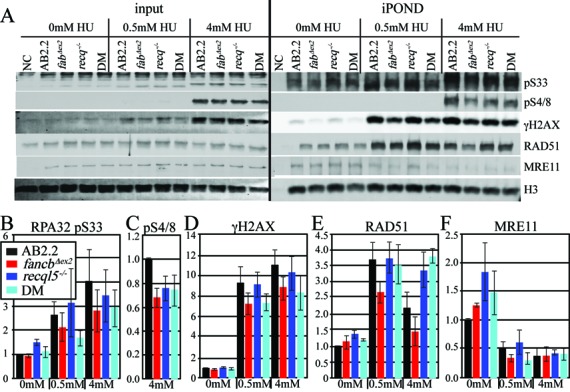
Protein levels purified at the nascent strand of the replication fork after 1.5-h exposure to 0.5-mM HU and 5-h exposure to 4-mM HU. (**A**) Western of input and iPOND purified proteins. (**B**) RPA32 pS33. (**C**) RPA32 p-S4/8. Only the 4-mM HU bands were calculated because signal was too low to quantify after exposure to 0- and 0.5-mM HU. (**D**) γH2AX. (**E**) RAD51. (**F**) MRE11. Quantitation of iPOND data: band intensity was measured, then normalized to H3 (loading control), then the normalized ratio (normalized protein intensity/normalized AB2.2 0 mM) was calculated. Standard error of the mean is shown for three to four experiments.

We used iPOND (isolation of proteins on nascent DNA) to purify replication protein A (RPA) near the nascent replication strand (17,47). RPA is a trimeric protein that binds single strand DNA at replication forks activating checkpoints after DNA polymerases and helicases are uncoupled (48). RPA phosphorylation distinguishes stalled from collapsed forks due to ATR phosphorylation of RPA32 S33 and DNA-PK_CS_ phosphorylation of RPA32 S4/S8, respectively (49). Cells were exposed to a mild HU condition that stalled forks (0.5 mM, 1.5 h) and a severe HU condition that collapsed forks (4 mM, 5 h) (40). As expected in AB2.2 control cells, mild and severe HU conditions increased RPA32 pS33 (Figure 7A and B) while only severe conditions increased RPA32 pS4/8 (Figure 7A and C). FANCB-deletion reduced RPA32 pS33 and RPA32 pS4/8 while RECQL5-deletion had no consistent impact (Figure 7A–C). Deletion of both proteins mimicked the FANCB-mutant. These results indicate that *fancb^Δex2^* cells have either fewer stalled/collapsed replication forks or the ATM/DNA-PK_CS_-responses are blunted. The latter is more plausible since FANCB-deletion increases breaks (Figure 2B and C) and impairs replication fork protection (Figures 3–5) and since the FA pathway interacts with DNA repair and DNA damage responses (50).

We used iPOND to observe γH2AX, RAD51 and MRE11 in control cells. γH2AX is generated in nucleosomes at stalled replication forks (47) and DNA DSBs (51), enables sister chromatid recombination (52) and helps recruit other proteins to damaged DNA (53). RAD51 forms a filament on single strand DNA to restart stalled forks (17) and to enable strand annealing with the sister chromatid to repair DSBs at collapsed forks as a member of the HR pathway (15). MRE11 is a 3′-5′ exonuclease that stabilizes the replisome (54), enables replication fork restart (55) and facilitates HR-mediated DSB repair through its exonuclease activity (56–58). For AB2.2 control cells, both mild and severe HU conditions increase the levels of γH2AX (Figure 7A and D) and RAD51 (Figure 7A and E) but decrease the levels of MRE11 (Figure 7A and F) similar to our past results (40).

We observed γH2AX, RAD51 and MRE11 in *fancb^Δex2^* cells, *recql5^−/−^* cells and *fancb^Δex2^ recql5^−/−^* cells. Deletion of FANCB, but not RECQL5, reduced γH2AX and RAD51 for both HU conditions (Figure 7A, D and E). The *fancb^Δex2^* cells also exhibit a small decrease in MRE11 at the mild HU condition (Figure 7A and F). Yet, RECQL5-deletion rescued levels of RAD51 in the *fancb^Δex2^* cells without rescuing levels of γH2AX and MRE11 suggesting RAD51 reduction is not due to lowered replication fork stress. This observation is in accord with RECQL5′s biochemical function of RAD51 filament disruption (25,26). Thus, RECQL5 dissociation of RAD51 filaments in FA-defective cells could suppress the potentially mutagenic events of RAD51-mediated replication fork restart and DSB repair (17,23) as seen after exposure to CPT (Figure 2C).

## DISCUSSION

The integration of RECQL5 and BLM with FANCB (and presumably the FA core complex) is lesion specific. In response to spontaneous damage RECQL5 and BLM enhanced chromatid breaks and radials, but suppressed isochromatid breaks. In response to MMC-induced damage, BLM caused chromatid breaks, isochromatid breaks and radials, while RECQL5 had little impact. In response to CPT-induced damage, RECQL5 suppressed chromatid breaks and radials while BLM had little impact. Thus, the integration of these RecQ helicases with the FA core complex is lesion specific.

**Figure 8.**
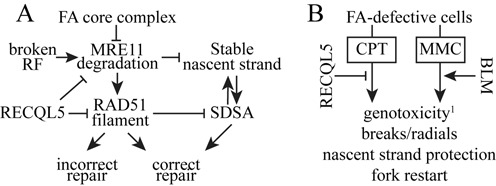
The functional interaction between RecQ helicases and the FA pathway. (**A**) A speculative model that proposes RECQL5 and the FA core complex suppresses chromosomal defects in an additive manner. (**B**) RECQL5 and BLM have different effects on FA-defective cells after exposure to CPT and MMC that could impact cancer treatment. (i) RECQL5 also suppressed cell death in *fancb^Δex2^* cells exposed to MMC.

In spite of this complexity, a speculative model starts to emerge. We hypothesize the FA core complex and RECQL5 stabilize replication forks in response to some lesions in a nonepistatic manner (Figure 8A). The following observations support such a model. (i) In FANCB-deleted cells, RECQL5 suppressed cell death and chromosomal breaks/radials during replication stress. (ii) In control cells, RECQL5 and FANCB protected the nascent replication strand from MRE11-mediated degradation and restarted stalled replication forks in a nonepistatic manner. (iii) FANCB facilitated a DNA damage response as measured by RPA32 phosphorylation and γH2AX. (iv) In FANCB-deleted cells, RECQL5 suppressed RAD51 levels at stalled and collapsed replication forks. Thus, RECQL5 and FANCB appear to stabilize replication forks to suppress chromosomal defects in a nonepistatic manner.

In FANCB-defective cells, RECQL5 could reduce unneeded RAD51 activity to enable chromosomal stability. RAD51-mediated HR has the potential to rearrange chromosomes (23). RECQL5 disrupts RAD51 filaments to enhance the use of SDSA (26), a less mutagenic pathway than RAD51-mediated HR. Therefore, it is possible that RECQL5 enhances the utilization of SDSA in FA-defective cells to cause better cell survival, fewer chromosomal rearrangements and reduced replication fork anomalies as compared to RAD51-mediated HR.

Oddly, this model predicts that for at least some types of DNA damage, the FA group 3 proteins that are members of RAD51-mediated HR (like BRCA2 and RAD51C) have a partially antithetical relationship with the FA group 1 proteins. This is possible since classification of the FA proteins is based on patient presentation, not protein function. Furthermore, the FA group 3 proteins do not participate in the quintessential FA activity of FANCD2 monoubiquitination. Thus, an antagonistic relationship between the FA group 1 and 3 proteins is possible. To further illustrate the complexity of the FA pathway, depletion of the mismatch repair protein MSH2 rescued MMC-hypersensitivity in cells defective for FANCJ (FA group 3) and FANCD2 (FA group 2), but not FANCA (FA group 1); thus, separating FA group 1 from groups 2 and 3 (59). Therefore, these FA groups do not always depend upon the other. Our data are consistent with a model that predicts the FA core complex stabilizes replication forks while RECQL5 disrupts RAD51 filaments to additively improve cell survival and chromosomal integrity.

The impact BLM has on the FA pathway is difficult to assess. Intuitively, BLM should suppress chromosomal defects as seen in control cells, yet BLM caused breaks and radials in FANCB-defective cells exposed to MMC, much like KU70 (8). It is possible that in the absence of the FA core complex, BLM caused toxic intermediate structures in response to MMC-induced damage, possibly as a member of BRAFT. Thus, we predict that the FA core complex maintains replication fork stability in a manner that suppresses BLM-mediated rearrangements after exposure to certain genotoxins like MMC.

The genetic interaction between FA and the RecQ helicases might be exploited for cancer therapy (Figure 8B). Our data show that RECQL5 suppresses replication fork defects, chromosomal breaks/radials in FA-defective cells exposed to CPT while BLM enhances cell death and chromosomal defects in FA cells exposed to MMC. Thus, RECQL5 and BLM have a divergent relationship with the FA core complex in response to replication fork stressors. Small molecules that inhibit RECQL5 might improve the effectiveness of replication fork blockers to treat FA-defective cancers. By contrast diminished BLM levels could indicate resistance to these agents leading to a poor therapeutic outcome. In the future, the role of RECQL5 and BLM should be tested in cells derived from FA-defective tumors to rigorously test the model presented in Figure 8B. Thus, the level of RECQL5 and BLM could be important when designing a therapeutic regime for FA-defective cancers.

## SUPPLEMENTARY DATA

Supplementary Data are available at NAR Online.

SUPPLEMENTARY DATA
