# Supplementary material for: RECQL5 and BLM exhibit divergent functions in cells defective for the Fanconi anemia pathway
Source: Nucleic Acids Res. 2014 Dec 17;43(2):893–903. doi: 10.1093/nar/gku1334 (PMC4333386; doi:10.1093/nar/gku1334)
Supplement: SUPPLEMENTARY DATA [file supp_43_2_893__index.html]

RECQL5 and BLM exhibit divergent functions in cells defective for the Fanconi anemia pathway — RECQL5 and BLM exhibit divergent functions in cells defective for the Fanconi anemia pathway — SUPPLEMENTARY DATA 

# RECQL5 and BLM exhibit divergent functions in cells defective for the Fanconi anemia pathway

## SUPPLEMENTARY DATA

**Files in this Data Supplement:**

- SUPPLEMENTARY DATA
- SUPPLEMENTARY DATA
