# Supplementary material for: RECQL5 and BLM exhibit divergent functions in cells defective for the Fanconi anemia pathway
Source: Nucleic Acids Res. 2014 Dec 17;43(2):893–903. doi: 10.1093/nar/gku1334 (PMC4333386; doi:10.1093/nar/gku1334)
Supplement: SUPPLEMENTARY DATA [file supp_gku1334_nar-02551-d-2014-File010.docx]

Supplementary Table 1. Statistics for nascent strand protection

| **Genotype** | **Agent** | **p-Value** |
| --- | --- | --- |
| AB2.2 v *fancb^Δex2^* | NT v NT | 4.5808E-10 |
| AB2.2 v *recql5^-/-^* | NT v NT | 0.0240 |
| AB2.2 v DM1 | NT v NT | 0.1246 |
| AB2.2 v *blm^-/-^* | NT v NT | 0.0014 |
| AB2.2 v DM2 | NT v NT | 0.0078 |
| *fancb^Δex2^* v *recql5^-/-^* | NT v NT | 0.0001 |
| *fancb^Δex2^* v DM1 | NT v NT | 8.2857E-14 |
| *fancb^Δex2^* v *blm^-/-^* | NT v NT | 3.901E-17 |
| *fancb^Δex2^* v DM2 | NT v NT | 1.6481E-16 |
| *recql5^-/-^* v DM1 | NT v NT | 0.0003 |
| *recql5^-/-^* v DM2 | NT v NT | 3.9666E-06 |
| *blm^-/-^* v DM2 | NT v NT | 0.4904 |
|  |  |  |
| AB2.2 v AB2.2 | NT v HU | 0.0202 |
| *fancb^Δex2^* v *fancb^Δex2^* | NT v HU | 1.1975E-85 |
| *recql5^-/-^* v *recql5^-/-^* | NT v HU | 3.9317E-73 |
| *blm^-/-^* v *blm^-/-^* | NT v HU | 0.5170 |
| DM1 v DM1 | NT v HU | 2.0513E-295 |
| DM2 v DM2 | NT v HU | 3.2428E-24 |
|  |  |  |
| AB2.2 v *fancb^Δex2^* | HU v HU | 8.9946E-221 |
| AB2.2 v *recql5^-/-^* | HU v HU | 5.2116E-136 |
| AB2.2 v DM1 | HU v HU | ~0 |
| AB2.2 v *blm5^-/-^* | HU v HU | 0.6052 |
| AB2.2 v DM2 | HU v HU | 7.1832E-25 |
| *fancb^Δex2^* v *recql5^-/-^* | HU v HU | 4.7238E-28 |
| *fancb^Δex2^* v DM1 | HU v HU | 2.1226E-108 |
| *fancb^Δex2^* v *blm^-/-^* | HU v HU | 6.2985E-183 |
| *fancb^Δex2^* v DM2 | HU v HU | 9.6995E-82 |
| *recql5^-/-^* v DM1 | HU v HU | 1.64047E-17 |
| *recql5^-/-^* v *blm^-/-^* | HU v HU | 2.9538E-112 |
| *recql5^-/-^* v DM2 | HU v HU | 5.8071E-32 |
| *blm^-/-^* v DM1 | HU v HU | 1.6725E-283 |
| *blm^-/-^* v DM2 | HU v HU | 2.3884E-23 |
|  |  |  |
| AB2.2 v AB2.2 | NT v CPT | 0.0064 |
| *fancb^Δex2^* v *fancb^Δex2^* | NT v CPT | 1.1291E-43 |
| *recql5^-/-^* v *recql5^-/-^* | NT v CPT | 2.3722E-135 |
| DM1 v DM1 | NT v CPT | 2.5795E-259 |
|  |  |  |
| AB2.2 v *fancb^Δex2^* | CPT v CPT | 5.325E-156 |
| AB2.2 v *recql5^-/-^* | CPT v CPT | 5.3336E-245 |
| AB2.2 v DM1 | CPT v CPT | ~0 |
| *fancb^Δex2^* v *recql5^-/-^* | CPT v CPT | 4.7238E-28 |
| *fancb^Δex2^* v DM1 | CPT v CPT | 2.122E-108 |
| *recql5^-/-^* v DM1 | CPT v CPT | 1.6405E-17 |
|  |  |  |
| AB2.2 v AB2.2 | mirin v HU + mirin | 0.21935 |
| *fancb^Δex2^* v *fancb^Δex2^* | mirin v HU + mirin | 0.69487 |
| *recql5^-/-^* v *recql5^-/-^* | mirin v HU + mirin | 0.99047 |
| DM1 v DM1 | mirin v HU + mirin | 0.03213 |

AB2.2, control cells

DM1, double mutant 1 (*fancb^Δex2^* *recql5^-/-^*)

DM2, double mutant 2 (*fancb^Δex2^* *blm^-/-^*)

Student T-test (for mean length)
