# Supplementary material for: RECQL5 and BLM exhibit divergent functions in cells defective for the Fanconi anemia pathway
Source: Nucleic Acids Res. 2014 Dec 17;43(2):893–903. doi: 10.1093/nar/gku1334 (PMC4333386; doi:10.1093/nar/gku1334)
Supplement: SUPPLEMENTARY DATA [file supp_gku1334_nar-02551-d-2014-File011.docx]

Supplementary Table 2. Statistics for replication fork restart

| **Genotype** | **NT v HU** |
| --- | --- |
| AB2.2 | 0.016 |
| *fancb^Δex2^* | 2.6732E-27 |
| *recql5^-/-^* | 3.68E-31 |
| DM1 | 1.635E-56 |
| *blm^-/-^* | 3.966E-33 |
| DM2 | 1.697E-19 |
|  | **HU v HU** |
| AB2.2 v *fancb^Δex2^* | 5.331E-18 |
| AB2.2 v *recql5^-/-^* | 1.623E-15 |
| AB2.2 v DM1 | 4.067E-47 |
| AB2.2 v *blm^-/-^* | 1.084E-15 |
| AB2.2 v DM2 | 1.579E-16 |
| *fancb^Δex2^* v *recql5^-/-^* | 0.2467 |
| *fancb^Δex2^* v DM1 | 1.914E-09 |
| *fancb^Δex2^* v *blm^-/-^* | 0.6786 |
| *fancb^Δex2^* v DM2 | 0.5946 |
| *recql5^-/-^* v DM1 | 1.214E-09 |
| *recql5^-/-^* v *blm^-/-^* | 0.4552 |
| *recql5^-/-^* v DM2 | 0.4857 |
| DM1 v DM2 | 3.319E-10 |
| DM1 v *blm^-/-^* | 5.523E-09 |
| *blm^-/-^* v DM2 | 0.963 |

AB2.2, control cells

DM1, double mutant 1 (*fancb^Δex2^* *recql5^-/-^*)

DM2, double mutant 2 (*fancb^Δex2^* *blm^-/-^*)

Yates-corrected chi-square test
